# Distribution Dynamics of Phthalate Esters in Surface Water and Sediment of the Middle-Lower Hanjiang River, China

**DOI:** 10.3390/ijerph19052702

**Published:** 2022-02-25

**Authors:** Lei Dong, Li Lin, Xiong Pan, Sheng Zhang, Zhanao Lv, Changqing Mi

**Affiliations:** 1 Basin Water Environmental Research Department, Changjiang River Scientific Research Institute, Wuhan 430010, China; dongleigushi@163.com (L.D.); panxiong@zju.edu.cn (X.P.); zhangsheng5453@163.com (S.Z.); lv1990@hust.edu.cn (Z.L.); michangqing789@163.com (C.M.); 2 Key Lab of Basin Water Resource and Eco-Environmental Science in Hubei Province, Wuhan 430010, China; 3 School of Chemistry and Chemical Engineering, Huazhong University of Science and Technology, Wuhan 430074, China

**Keywords:** phthalate esters, water, sediments, Hanjiang River, distribution, suspended sediment

## Abstract

Phthalate esters (PAEs) are endocrine-disrupting chemicals that pose potential risks to human health. Water and sediments are crucial carriers and storage media for the migration and transformation of PAEs. In this study, six congeners of PAEs were measured in water and sediment samples to elucidate their spatial distribution, congener profiles, and ecological risks in the middle-lower Hanjiang River during the wet and dry seasons. The concentration of the Σ_6_PAEs ranged from 592 to 2.75 × 10^3^ ng/L with an average of 1.47 × 10^3^ ng/L in surface water, while the concentration of the Σ_6_PAEs ranged from 1.12 × 10^3^ to 6.61 × 10^3^ ng/g with an average of 2.69 × 10^3^ ng/g in sediments. In general, PAE concentrations were ranked as sediment > water, and dry season > wet season. DEHP and DBP were the dominant PAEs in the middle-lower Hanjiang River in surface water and sediments. SPSS analysis showed that dissolved organic carbon (DOC) in surface water was significantly correlated with the concentration of DBP, DEHP, and the ∑_6_PAEs, while organic matter (OM) was significantly correlated with the concentration of the ∑_6_PAEs in sediments. The concentrations of PAEs were irregularly distributed and varied significantly in surface water and sediments. Compared with other regions at home and abroad, the pollution levels of surface water and sediments in the middle-lower Hanjiang River were relatively low and not enough to have a negative impact on the local water’s ecological environment. However, the supervision of land-based discharge should still be strengthened.

## 1. Background

Phthalate esters (PAEs) are a group of chemical compounds that are widely used as nonreactive plasticizers not only in polyvinyl plastics (PVC) but also in a broad range of industrial processes and consumer products, including cosmetics, building materials, insect repellents, automobile parts, and food packaging [1]. PAEs are easily released into the environment during the processes of manufacturing and application via evaporation and leaching from domestic and industrial effluents [2,3,4,5]. Global PAE production exceeds 8.0 million tons annually, and it has been reported that PAE consumption in 2011 reached approximately 2.2 million tons in China [6]. Previous studies have shown that PAEs are endocrine-disrupting chemicals that pose potential health risks to humans and other organisms; for example, they can disrupt the hormonal balance of mammalian species [7]. Six PAE monomers are listed as priority control pollutants by the European Union (EU) and the Environmental Protection Agency in the United States (USEPA) [4]. PAE-containing products in industry and households have led to PAEs being ubiquitous in various environments, including air, water, sediments, soil, and food [7,8,9].

Water and sediments are important carriers and storage media for the migration and transformation of PAEs [10]. The distribution characteristics of PAEs in various environmental media can reflect their pollution levels and their potential impacts on the health of aquatic ecosystems [5,11]. Sediment in aquatic ecosystems can significantly absorb PAEs and play a key role in the migration, transformation, and purification of PAEs [4,7]. After PAEs’ exposure to water, they can be adsorbed, complexed, flocculated, and precipitated by suspended sediment particles and finally accumulated in the form of sediment [11,12].

With a length of 1567 km, the main stream of the Hanjiang River flows through the Shanxi and Hubei provinces and finally discharges into the Yangtze River in Wuhan; it is the largest tributary of the Yangtze River [12]. The middle-lower Hanjiang River, with a total length of 652 km, starts at the Danjiangkou Dam and ends at the Yangtze River, accounting for 41.3% of the total length of the Hanjiang River [13,14]. The middle-lower Hanjiang River is one of the most economically viable areas in Hubei Province. It is the main base of agricultural commodities, the automobile industry corridor, the equipment-manufacturing industry, textiles, and clothing production, as well as the main source of fresh drinking water for approximately 7.73 million people living in several cities and an important source of edible aquatic products in Hubei Province [15]. The Danjiangkou Reservoir is located in the upper reach of the Hanjiang River and serves as a water resource for the middle line of China’s South-to-North Water Diversion Project. Since the operation of the middle line of China’s South-to-North Water Diversion Project began on December 12, 2014, conflicts between the economic development and ecological protection of the Hanjiang River Basin have become more prominent. The water purification capacity of the middle-lower Hanjiang River had been reduced due to the reduction in upland water [14]. Elevated environmental pressures such as the outbreak of diatom blooms [16,17] and the severe pollution of tributary water quality in the middle-lower Hanjiang River [14] have threatened the biological productivities of Hanjiang River ecosystems during the last few decades; consequently, much research and management work has been carried out. Recently, some organic contaminants, such as OCPs, PCBs, PBDEs, and PAHs, were found in the surface water and sediments of the middle-lower Hanjiang River [13,18], which indicated that synthetic organic chemicals might be an emerging concern for the ecosystem of the middle-lower Hanjiang River.

It should be noted that the analysis of PAEs in surface water and sediment is essential and highly important, and studies reporting the levels of PAEs are necessary for policy makers and for monitoring purposes of the middle-lower Hanjiang River. Analyzing the literature, we found that Hang et al. (2007) reported that dimethyl phthalate (DMP) and di-n-butyl phthalate (DBP) in the surface water of the Hanjiang River were 106 and 456 ng/L, respectively, and the concentrations of other PAE monomers were unknown. Liu et al. (2010) reported 16 PAE concentrations in the topsoil ranging from 253 to 2.52 × 10^3^ ng/g, with an average value of 927 ng/g, and the study area was located in the Jianghan Plain, which belongs to the middle-lower Hanjiang River [19]. However, considering the background of intensified human activities and great changes in the hydrological situation, there is no systematic report on the environmental levels and ecological risks of PAEs in the middle-lower Hanjiang River.

Obviously, more research is necessary to provide reliable data for carrying out a risk assessment of PAEs in surface water and sediments along the middle-lower Hanjiang River, considering that PAEs are toxic, bioaccumulative, and detected with high frequency. In this study, samples of surface water and sediments were collected from the urban river section, natural river section, and the lower reaches of the dam in the middle-lower Hanjiang River, combined with the analysis of historical literature. The objectives were to: (1) determine the pollution levels and characteristics of PAEs in the surface water and sediments along the middle-lower Hanjiang River in dry and wet seasons; (2) perform an environmental risk assessment for PAEs in the surface water and sediments in the middle-lower Hanjiang River; and (3) preliminarily elaborate the reasons for underlying differences in PAE distributions in environmental media regarding pollution sources, sediment adsorption, and water–sediment relationships. Further, this work provides the first documentation of surface water and sediment contamination by PAEs in the middle-lower Hanjiang River, thereby enabling a monitoring program to study the pollution status and changing trends of PAEs over space and time, which is also beneficial for water environmental management and protection in the economic belt of the Hanjiang River.

## 2. Methods

### 2.1. Reagents and Chemicals

All solvents, including hexane, methylene chloride, acetone, methanol, and ethyl acetate, which had purity levels of at least 99%, were of HPLC grade and obtained from Fisher Chemical Co. (Fair Lawn, OH, USA). Six PAE standard solutions used in this study including dimethyl phthalate (DMP), diethyl phthalate (DEP), di-n-butyl phthalate (DBP), butyl benzyl phthalate (BBP), di-2-Ethylhexyl phthalate (DEHP), and di-n-octyl phthalate (DNOP) were supplied by AccuStandard Inc. (New Haven, CT, USA, 99%). Solid-phase extraction membranes (ENVI-18 DSK) were obtained from Sigma-Aldrich Chemistry Co. (St. Louis, MO, USA). Anhydrous sodium sulfate (analytical grade, Beijing Chemical Reagent Co., Shanghai, China) was baked in a furnace oven (FP-40, China) at 550 °C for 5 h to remove any organics or water, then kept in a sealed desiccator prior to use. Water was prepared from a Milli-Q system (Millipore; Bedford, MA, USA). Glassware was cleaned with acetone first and then n-hexane.

### 2.2. Study Area

**Figure 1:**
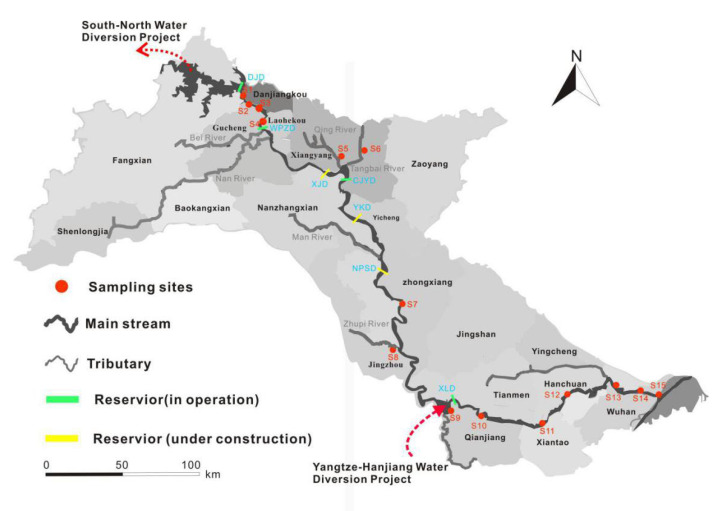
Map showing sampling sites in the middle-lower Hanjiang River. DJD: Danjiangkou Dam, WPZD: Wangpuzhou Dam, XJD: Xinji Dam, CJYD: Cuijiaying Dam, YKD: Yakou Dam, NRSD: Nianpanshan Dam, XLD: Xinglong Dam.

The sampling sites along the middle-lower Hanjiang River are plotted in Figure 1. Detailed information is listed in Table A1. Water and sediment samples were collected from the middle-lower Hanjiang River in June 2019 (wet season) and in January 2020 (dry season).

Downstream of the Danjiangkou Reservoir, six cascade reservoirs are located (as shown in Figure 1), of which Wangpuzhou Reservoir, Cuijiaying Reservoir, and Xinglong Reservoir are in operation, while the other three reservoirs, Xinji Reservoir, Yakou Reservoir, and Nianpanshan Reservoir, are still under construction.

### 2.3. Sample Collection

All surface water samples (about 20 cm below the water’s surface) were sealed in 5 L glass containers and carried back to the laboratory within the day and stored at 4 °C in a refrigerator before further research. On the same day, 4 L surface water was passed through a 0.45 μm glass fiber membrane at the laboratory, and then the water sample was passed through a methanol-activated solid-phase extraction membrane (SPE) to isolate the PAEs [20,21]. Finally, the solid-phase extraction membrane that contained the PAEs was wrapped in foil and then refrigerated for further analysis. The other 1 L from the initial 5 L water sample was obtained to analyze the concentrations of dissolved organic carbon (DOC), total nitrogen (TN), total phosphorus (TP), and ammonia nitrogen (NH_3_-N). Moreover, the pH, dissolved oxygen (DO), temperature (T), and oxidation–reduction potential (ORP) were field-measured with a multiparameter water quality probe (USA, YSI EXO2).

The surface sediments (0–5 cm) from 15 sampling sites were collected with stainless steel Peterson grab samplers and put into a glass petri dish. The samples were stored in ice on their way to the laboratory, where they were stored at −20 °C until further processing. Each sample was divided into two sub-samples: one sub-sample was thawed for TN, TP, and organic matter (OM) analysis, the other sub-sample was freeze-dried and then carefully homogenized over 100 mesh for PAE analysis in sediments.

### 2.4. Sample Analysis

#### 2.4.1. The Extraction Process of Water and Sediment

Refer to the extraction methods of our previous study [22]. For water samples, SPE extraction disks were eluted twice with ethyl acetate and dichloromethane-ethyl acetate (1:1, *v*:*v*). Then, the extracts were combined and dried over anhydrous sodium sulphate. The extracts were concentrated to 0.1 mL in a rotary evaporator (R-210, Buchi, Flawil, Switzerland) and a thermovap sample concentrator (N-EVAP, Organomation, Berlin, MA, USA). Finally, the samples were redissolved in 1 mL of n-hexane for analysis.

The sediment was lyophilized by a freeze dryer (FD5-series, SIM, Newark, DE, USA) for 72 h and then crushed and passed through a 100-mesh sieve. Approximately 2 g of the dry sample was accurately weighed and then transferred to a 50 mL extract tube containing 25 mL of acetone–hexane (1:1, v:v) organic extraction solvent. Each mixture was extracted with a microwave digestion system (Mars6, CEM, Matthews, NC, USA) wherein the temperature changed at a rate of 10 °C/min from room temperature to 120 °C; the 120 °C temperature was maintained for 30 min. After the microwave extraction was completed, the supernatant was passed through anhydrous sodium sulphate. Then each sample was extracted and purified in a Florisil SPE Cartridge (1 g, 6 mL/30 pcs). Finally, the eluent from the Florisil SPE Cartridge was evaporated to 0.1 mL using a rotary evaporator and nitrogen flow, and then re-dissolved in 1 mL of n-hexane for measurement.

#### 2.4.2. GC/MS Analysis

Analytical detection of PAEs was performed using a gas chromatography–mass spectrometer (GC–MS) (7890B/5977A, Agilent, Palo Alto, CA, USA) equipped with a DB-5 ms fused silica capillary column (30 m × 0.25 mm × 0.25 μm, Agilent, Palo Alto, CA, USA). High-purity helium (99.999%) served as the carrier gas at a flow rate of 1.0 mL/min. The transfer line and the inlet temperature were maintained at 300 and 250 °C, respectively. The injection volume was 1.0 μL, and the splitless injection mode was used. The oven temperature program for the PAEs was as follows: initial temperature of 70 °C held for 2 min; increased to 130 °C at 20 °C/min; increased to 200 °C at 5 °C/min; and increased to 300 °C at 15 °C/min, held for 5 min [22].

#### 2.4.3. Quality Control

Quality control and quality assurance in the sample analyses were implemented according to the regulations for water environmental monitoring of China (SL 219-2013). The quantitative standards for PAEs in the samples were determined using a combination of external standard methods. The linearity correlation coefficients were between 0.999 and 1.00 for the 6 PAE monomers. The limits of detection (LOD) were determined based on the concentrations that existed at three times the signal-to-noise ratio. The LOD of the water samples ranged from 0.120 to 0.920 ng/L, whereas the LOD of the sediment samples ranged from 0.250 to 1.85 ng/g [22]. The recoveries of 6 PAE monomers for the water samples were 86.9–110%, while the recoveries of 6 PAE monomers for the sediment samples were 63.9–75.5%. In total, 6 PAE monomers were quantitatively analyzed using Mass Hunter data acquisition software on an Agilent 7890B/5977A GC/MS instrument. To avoid the injection contamination caused by the analysis process, a sample blank was made after every 10 samples analyzed. The sample blank value was below LOD. Any datum that was under the LOD was calculated as not detected (ND) [23].

### 2.5. Data Collection and Processing

Hydrologic and sediment data were collected from the Changjiang River Sediment Bulletin [24]. All the figures were constructed using the software Origin (version 2019) and Surfer (version 16.0). Pearson correlation analysis of PAEs, TP, CODMn, Chl-*a*, TN, NH_4_-N, DOC, DBP, and DEHP (data normally distributed) in surface water and Pearson correlation analysis of PAEs, OM, TN, TP, DBP, and DEHP (data normally distributed) in sediments were carried out using SPSS (IBM SPSS Statistics 22.0).

## 3. Results

### 3.1. Occurrences, Spatial Distribution, and Pollution Levels

The concentrations of the 6 USEPA priority PAEs were measured in all the water and sediment samples from the wet season and the dry season. The detection frequencies of the predominant PAE monomers were as follows: DMP: 80.0%, DEP: 100%, DBP: 100%, BBP: 76.6%, DEHP: 100%, and DNOP: 86.6% in the water samples, and DMP: 100%, DEP: 100%, DBP: 100%, BBP: 86.6%, DEHP: 100%, and DNOP: 90.0% in the sediment samples.

#### 3.1.1. Surface Water

The exposure concentrations of the six PAE monomers in the middle-lower Hanjiang River are presented in Table A2, and detailed information is listed in Table A3. The total concentrations of the six PAE monomers in the water samples were detected in the range of 592–2.75 × 10^3^ ng/L (the mean value was 1.47 × 10^3^ ng/L). The PAE concentrations indicated significant seasonal variation. DBP and DEHP in the surface water samples of the middle-lower Hanjiang River did not exceed the standard limits of 3.00 × 10^3^ and 8.00 × 10^3^ ng/L, respectively, which are based on the National Surface Water Environmental Quality Standards (GB3838-2002) in China. The exposure concentrations of the ∑_6_PAEs in the wet season were much lower than those in the dry season, with the mean concentration of DEP, DBP, BBP, DEHP, and DNOP in the dry season about 1.20–5.61 orders of magnitude higher than that in the wet season. This may be because the rainfall and the high-speed flow in the river acted as diluents during the wet season, improving its self-purification ability [25]. In addition, during the wet season, the volume of the water body increases significantly, thus diluting the concentrations of PAEs [26]. Similar seasonal changes have occurred in the main stream of the Yangtze River [25] and Poyang Lake [27].

**Figure 2:**
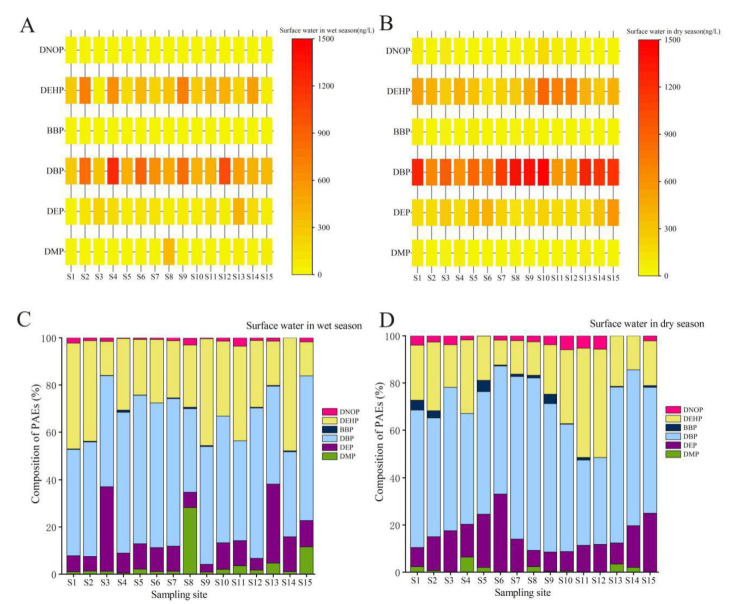
Distribution and composition of the ∑_6_PAEs in the surface water of the middle-lower Hanjiang River. (**A**) distribution of the ∑_6_PAEs in the wet season; (**B**) distribution of the ∑_6_PAEs in the dry season; (**C**) composition of the ∑_6_PAEs in the wet season; (**D**) composition of the ∑_6_PAEs in the dry season.

The distributions of the six PAE monomers in different seasons are shown in Figure 2A,B. Combined with monitoring data results, the exposure concentrations of PAEs in different sampling sites exhibited significant differences in the wet season and dry season. Higher PAE concentrations were mainly concentrated in urban areas and agricultural planting areas, for example, the Danjiangkou urban section (S2), Laohekou urban section (S4), downstream of the Xinglongba gate (S9, agricultural planting area), and the Qianjiang urban section (S10), while the concentration of PAEs in other sampling sites was relatively low. These sites with higher PAE concentrations were mostly influenced by human activities, inevitably receiving household wastes and industrial wastewater containing PAEs from surrounding residents, chemical reagent factories, or fine chemical mills.

The relative composition profiles of PAEs in the surface water samples from the middle-lower Hanjiang River were different depending on the sampling locations (Figure 2C,D). Generally, DBP and DEHP dominated, with an average contribution of up to 60.0% of the ∑_6_PAEs in the wet season or dry season. Almost one-half of the sampling sites showed that DBP was the dominant compound with a relative contribution of >50.0% to the ∑_6_PAEs, while DEHP dominated at one-half of the sampling sites, accounting for >20.0% of the ∑_6_PAEs in the wet season or dry season. The most abundant PAE in the water samples was DBP, accounting for 35.3–72.9% of the ∑_6_PAEs, followed by DEHP (10.6–47.9%). DBP and DEHP were the most prevalent PAEs in the water body, which was consistent with those found in the Wuhan Section of the Yangtze River [28,29] and the Yangtze River Estuary in a previous study [30].

The water pollution levels of the middle-lower Hanjiang River were compared with the pollution levels of other rivers in China and abroad (Table A4). The mean concentrations of the ΣPAEs in the middle-lower Hanjiang River were approximately 4 times higher than those in the Three Gorges Reservoir [22] and were approximately 1.6 times higher than those in the Jiangsu section of the Yangtze River [31] and the Changjiang River Estuary [32]. However, the mean concentrations of the ΣPAEs in the middle-lower Hanjiang River were about 4 and 13 times lower than those in the Jiulong River [33] and the Songhua River [9], separately. For the main PAE monomers, the maximum DBP measured in the middle-lower Hanjiang River (1.48 × 10^3^ ng/L) was about 10–20 times lower than that in the Songhua River (11.8 × 10^3^ ng/L) [9], and the mid-lower reaches of the Yellow River (26.0 × 10^3^ ng/L). DBP was commonly recorded in the Kaveri River from India (250 ng/L) [2] and the Jiangsu section of the Yangtze River (max. value 286 ng/L) [31], approximately five times lower than that in the middle-lower Hanjiang River in this study. The pollution levels of DEHP in the middle-lower Hanjiang River were 10–20 times lower than those of rivers in China and abroad, such as the Humber River [34], the mid-lower reaches of the Yellow River, the Songhua River [9], and the Jiulong River [33]. Meanwhile, the maximum pollution level of DEHP in the middle-lower Hanjiang River was approximately 2 times higher than that in the Three Gorges Reservoir [22]. Overall, compared with the levels in other rivers in China and abroad, the pollution levels of the ΣPAEs in surface water were relatively low along the middle-lower Hanjiang River.

#### 3.1.2. Sediments

The exposure concentrations of DMP, DEP, DBP, BBP, DEHP, and DNOP in the sediments of the middle-lower Hanjiang River are presented in Table A5. The concentrations of the ∑_6_PAEs in the sediments ranged from 1.12 × 10^3^ to 6.61 × 10^3^ ng/g (with a mean value of 2.69 × 10^3^ ng/g). The ranges of DMP, DEP, DBP, BBP, DEHP and DNOP concentrations in the sediments were 2.30–190 ng/g, 2.00–620 ng/g, 159–4.33 × 10^3^ ng/g, ND–21.1 ng/g, 341–1.89 × 10^3^ ng/g, and ND–416 ng/g, respectively. The average concentrations of the six PAE monomers followed the order: DEHP (1.11 × 10^3^ ng/g) > DBP (920 ng/g) > DNOP (140 ng/g) > DEP (132 ng/g) > DMP (10.8 ng/g) > BBP (5.40 ng/g) in the wet season, whereas DBP (2.01 × 10^3^ ng/L) > DEHP (735 ng/L) > DEP (196 ng/L) >DNOP (63.8 ng/L) > DMP (49.3 ng/L) > BBP (5.90 ng/L) were the average concentrations in the dry season. The exposure concentrations of the ∑_6_PAEs in the wet season were much lower than those in the dry season. Similar to the results of the water samples, DBP and DEHP were also most frequently detected in sediment samples with 100% detection frequencies and the highest mean contamination levels of the six PAE monomers. This result was consistent with the findings in previous studies [22,28,29,35] where DBP and DEHP were also the dominant components in sediments.

**Figure 3:**
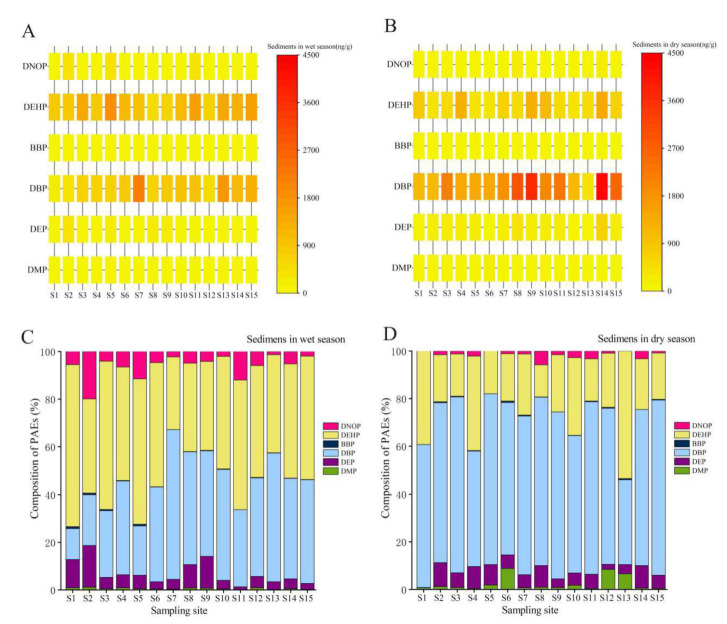
Distribution and composition of the ∑_6_PAEs in the sediments of the middle-lower Hanjiang River. (**A**) distribution of the ∑_6_PAEs in the wet season; (**B**) distribution of the ∑_6_PAEs in the dry season; (**C**) composition of the ∑_6_PAEs in the wet season; (**D**) composition of the ∑_6_PAEs in the dry season.

In general, the distribution trend of the ΣPAEs in the sediments of the middle-lower Hanjiang River in the wet season was not consistent with that in the dry season (Figure 3A,B). However, in the middle-lower Hanjiang River, the higher concentrations of PAEs in the sediments were mainly concentrated in urban river sections, such as the Zhupi River (S8), downstream of the Xinglongba gate (S9), and Wuhan city urban area (S13, S14, and S15), which were likely caused by a stronger influence of urban activities, indicating that PAEs mainly came from point source pollution.

The compositions of six PAEs in different seasons are illustrated in Figure 3C,D. Similar to those in the water samples, DBP and DEHP dominated, with an average contribution of up to 85.0% of the ∑_6_PAEs, which was consistent with those found in the Wuhan Section of the Yangtze River [28,29] and the Yangtze River Estuary [32] in a previous study. Specifically, the results showed that DBP and DEHP were the major components of PAEs in the surface water of the middle-lower Hanjiang River with a proportion of 60.7–95.3% (average: 86.7%) in the wet season and 83.7–99.2% (average: 89.8%) in the dry season. Following DEP, DNOP contributed to the total PAE concentration by 1.21–19.9% (average: 6.30%) in the wet season and 0.100–10.1% (average: 3.90%) in the dry season. The concentrations of PAEs were irregularly distributed and varied significantly along the middle-lower Hanjiang River in surface water and sediments, and the patterns of different distributions of PAEs varied substantially along the river without clear trends in surface water and sediments (Figure 2 and Figure 3).

The sediment pollution levels of the middle-lower Hanjiang River were compared with the pollution levels of other rivers in China and abroad (Table A4). The mean concentrations of the ΣPAEs in the sediments found in the middle-lower Hanjiang River were approximately 4.80–6.80 times lower than those at areas such as the Changjiang River Estuary [32] and the Songhua River [35] and were approximately equal to those of areas such as Cochin estuary [4] and the Three Gorges Reservoir [22] and remarkably higher than that in the Xijiang River [36]. In general, the ΣPAEs’ sediment pollution levels were low in the middle-lower Hanjiang River.

### 3.2. Physiochemical Characteristics of Water and Sediment

#### 3.2.1. Surface Water

**Figure A1:**
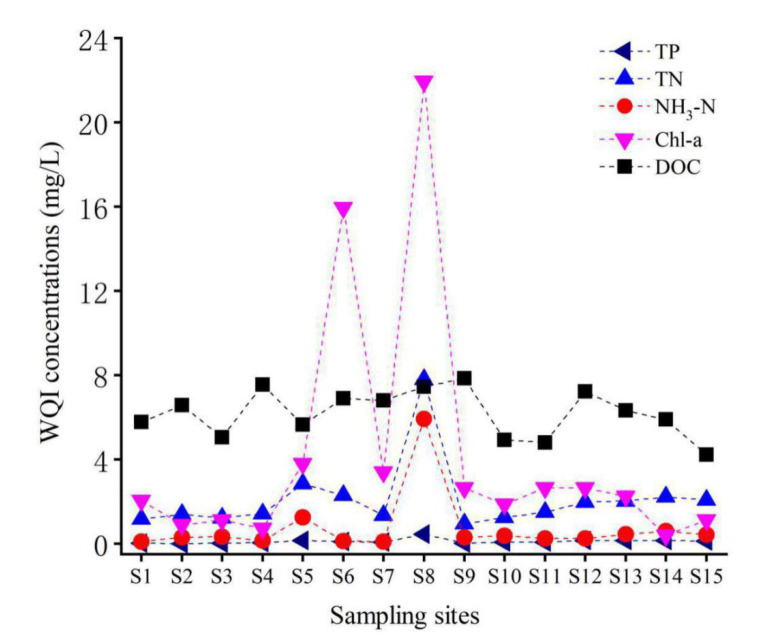
Concentration distribution trend of the water quality index (WQI) in the wet season from the middle-lower Hanjiang River.

It has been reported that the environmental behavior and fate of PAEs are affected by their physical and chemical properties as well as the characteristics of water and sediments [10,37]. Figure A1 shows the trends of conventional water quality indexes (WQIs) measured in the middle-lower Hanjiang River. It can be seen that the WQIs involved in this study display similar trends along the middle-lower Hanjiang River (including TP, Chl-*a*, TN, NH_3_-N). During the wet season, TP concentration at the various sections ranged between 0.01 mg/L and 0.46 mg/L. Chl-*a* in the Zhupi River (S8) was higher than that in other sections. The average TN concentration was 2.10 mg/L, with the highest value also being observed at the Zhupi River (7.80 mg/L). The NH_3_-N concentration among the sections varied considerably; the lowest value was observed at the Danjiangkou Reservoir (S1, 0.09 mg/L), whereas the highest was obtained at the Zhupi River (5.91 mg/L). DOC concentration did not vary substantially among the sections (mean: 6.20 mg/L).

In addition, TP, Chl-*a*, TN, and NH_3_-N showed Pearson correlations to each other to some extent, but their Pearson correlation with PAEs was not significant (Table A6). However, the concentration of DBP, DEHP, and the ∑_6_PAEs in surface water was significantly positively correlated (*p* < 0.01) with DOC, with Pearson correlations indexes of 0.737, 0.647, and 0.814, respectively. DOC refers to the water-soluble organic matter that can pass through 0.450 μm microporous membranes, mainly including toxic organic pollutants such as PAEs. As a result, it was indicated that the concentration of DOC had significant effects on the concentration of DBP, DEHP and PAEs.

#### 3.2.2. Sediments

**Figure A2:**
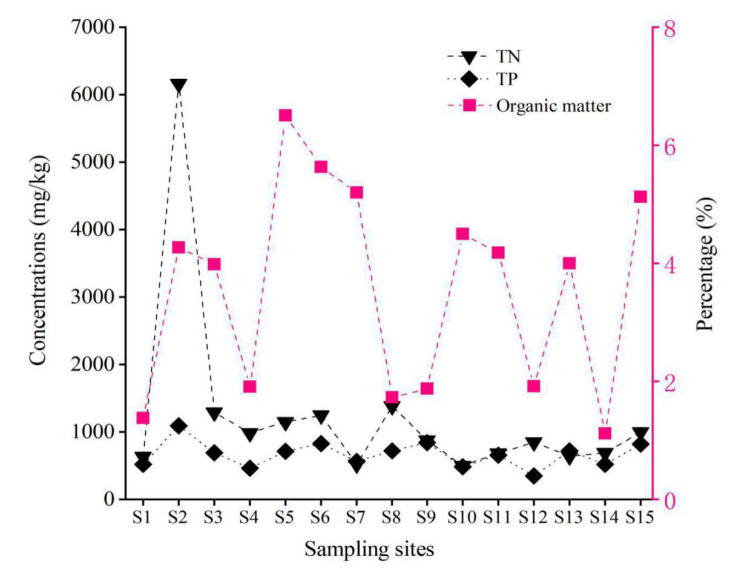
Concentration distribution trend of TN, TP, and organic matter in sediments in the wet season from the middle-lower Hanjiang River.

The TN concentrations ranged from 498 to 6.16 × 10^3^ mg/kg, and the TP concentrations of the sediments were between 352 mg/kg and 1.10 × 10^3^ mg/kg, with averages of 1.24 × 10^3^ and 667 mg/kg, respectively (Figure A2). The organic matter (OM) concentrations in sediment ranged from 1.12 to 6.51%, with an average of 3.56%.

**Figure A3:**
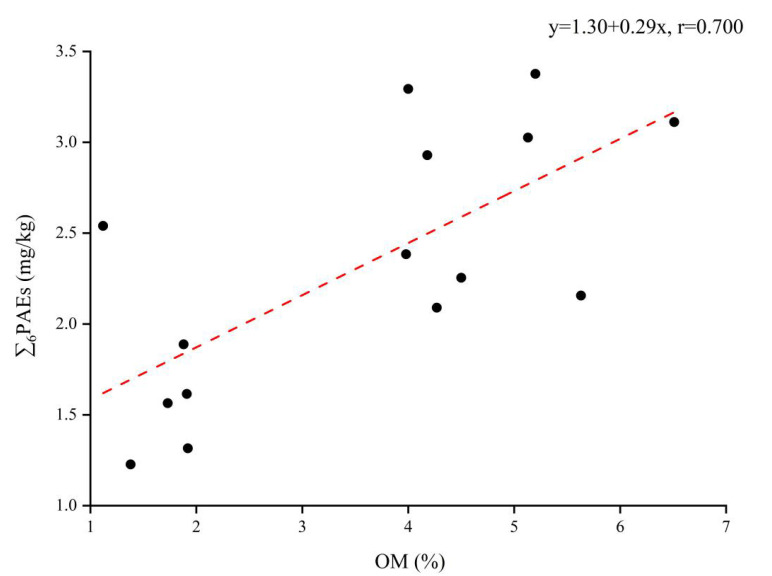
Correlation coefficients between the ∑_6_PAEs and OM of sediments in the wet season from the middle-lower Hanjiang River.

**Figure A4:**
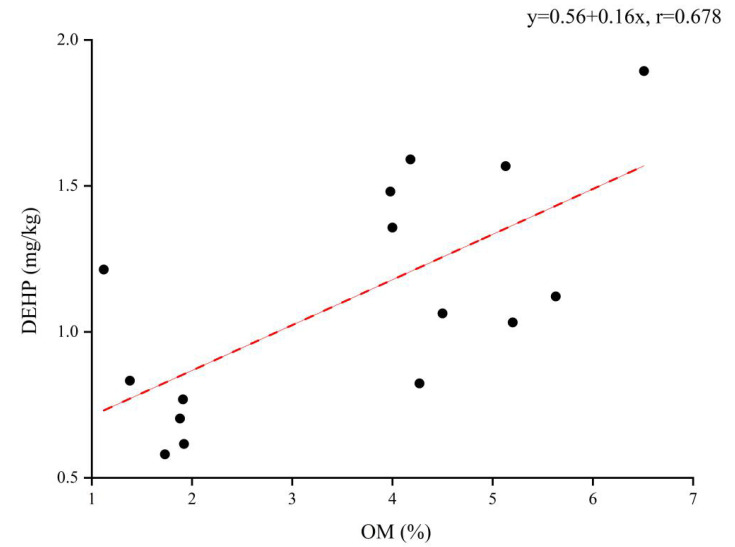
Correlation coefficients between DEHP and OM of sediments in the wet season from the middle-lower Hanjiang River.

There were no significant Pearson correlations (*p* < 0.01) between PAEs and TN and PAEs and TP in sediments (Table A7), indicating that TN and TP were not factors controlling the behavior of PAEs in sediments. However, significant positive (*p* < 0.01) correlation coefficients were observed between the ∑_6_PAEs and the sediments’ OM concentrations (y = 1.30 + 0.29x, r = 0.700) and between DEHP and the sediments’ OM concentrations (y = 0.56 + 0.16x, r = 0.678) (Figure A3 and Figure A4). The results show that OM could impact the concentrations of ∑_6_PAEs in sediments. Because of the hydrophobic characteristics of the ∑_6_PAEs, they may be readily adsorbed and fixed by the precipitate OM in sediments.

## 4. Discussion

### 4.1. Ecological Risk Assessment

#### 4.1.1. Surface Water

Based on toxicological data and numerical calculations, the USEPA has established ambient water quality criteria for human health (USEPA, 2015), which provide guidance for states to use to establish water quality standards and ultimately provide a basis for controlling discharges or releases of PAEs.

As shown in Table 1, the human health ambient water quality criteria (AWQC) represents the maximum acceptable levels of pollutants in a water body that are not expected to cause adverse effects on human health by consumption of “water + organism” or “organism only”. In this study, the concentrations of DMP, DEP, DBP, and BBP in all surface water samples from the middle-lower Hanjiang River were lower than those of the human health AWQC for the consumption of “water + organism” or “organism only” in the wet season and the dry season, which indicated a low risk to human health (Table 1).

However, more than half of the sampling sites (53.3%) showed levels of DEHP that exceeded the value of the human health AWQC for the consumption of “water + organism”, while 46.7% of the sampling sites exceeded the value of the human health AWQC for the consumption of “organism only” in the wet season (Table 1 and Table A3). Further, 60.0% of the sampling sites showed levels of DEHP that exceeded the value of the human health AWQC for the consumption of “water + organism” and “organism only” in the dry season (Table 1 and Table A3). In particular, DEHP levels were relatively high (more than 600 ng/L) at several sampling sites of the middle-lower Hanjiang River, such as the Danjiangkou urban section (S2), Laohekou urban section (S4), and downstream of the Xinglongba gate (S9) in the wet season, and the Qianjiang urban section (S10) and Hanchuan urban section (S12) in dry season. Therefore, there might be a potential impact of DEHP on human health in these areas.

#### 4.1.2. Sediments

Sediment accumulates PAEs in the process of water adsorption. When different PAE monomers coexist, there may be competition in the adsorption process, and when the environmental redox conditions change, it will also lead to the release of PAEs in the sediment [38,39]. This study shows that the order of PAE concentration is sediment > water (Table A2 and Table A5), which indicates that sediment is an important reservoir of PAEs. These contaminants might cause adverse ecological impacts. There have been some studies on the impact assessment of the ecological risks of PAEs [7,8,23,35,40,41,42]. However, no unified standards have been established for the assessment of the ecological risks of PAEs in sediments.

Regarding the environmental risk levels (ERLs) of the two PAE monomers determined [23,41], the ERLs of DBP and DEHP are 700 ng/g and 1.00 × 10^3^ ng/g, respectively. When the relative pollution factor (RCF = C_PAEs_/ERLs, C_PAEs_ is the measured environmental concentration of PAEs) is less than 1, PAE concentrations do not cause endocrine disruption and ecotoxicity risks; when the RCF is greater than 1, PAE concentrations can cause endocrine disruption and ecotoxicity risks. In the present study, both DBP and DEHP concentrations in 60% of the sediment samples exceeded the ERLs in the wet season, while DBP concentrations in 93.3% samples and DEHP concentrations in 26.6% samples exceeded the ERLs in the dry season (Table A5 and Figure 3). It should be noted that both DBP and DEHP concentrations of the sediment samples at some sampling sites exceed the ERLs, such as those in the Qianjiang urban section (S10) and midstream of Wuhan city (S14) in the wet and dry seasons. These results indicated that DBP and DEHP might exhibit potential risks to aquatic life and human health in some urban river sections of the middle-lower Hanjiang River, which were similar to the results of some other river studies in China [1,33].

For the other PAE monomers (including DMP, DEP, BBP, and DNOP), this study used the sediment management standards from the Washington State Department of Ecology, USA, each of whose values is 610 ng/g [33]. Therefore, DMP, DEP, BBP, and DNOP did not exceed the sediment quality standard limit, indicating that the ecological risks of DMP, DEP, BBP, and DNOP were relatively low.

### 4.2. Source Analysis

As for surface water, DBP, DEHP, DMP, and DEHP are found in high concentrations in household wastes (e.g., toys, plastic containers, and commodities) and heavy chemical industries [30]. DEP was the predominant PAE monomer used in cosmetic and personal care products [43]. In sediments, DBP mainly came from heavy chemical industries [30]. In addition, DEHP is the major PAE that leaches from household wastes [44] and is also a major component in atmospheric particles [45]. Recently, PAEs have been found in PM2.5 and PM10 in the ambient air of the Wuhan, Xiangyang, and Xiantao cities [46,47,48,49]. Moreover, atmospheric transport and deposition are also important sources of organic contaminants in the sediment environment (Mi et al., 2019). Therefore, long-range atmospheric transport deposition might also be a source of DEHP in the sediments of the middle-lower Hanjiang River, which requires further and ongoing research in the future. On the whole, domestic waste and heavy chemical industries might be the input sources for PAEs in sediments of the middle-lower Hanjiang River, but this still requires follow-up systematic research.

### 4.3. Impact of Suspended Sediment on the Behavior of PAEs

Owing to the hydrophobicity of organic toxic pollutants such as PAEs, they are easily adsorbed on suspended sediment (SS) in water bodies; therefore, the distribution dynamics of PAEs in different environmental media are not only related to pollution source discharge and sediment adsorption, but also related to SS concentrations and their transport and transformation characteristics [32,50,51].

The South-to-North Water Diversion Project diverts water from the Danjiangkou Reservoir and transports water to Beijing and Tianjin. The middle route of the South-to-North Water Diversion Project is divided into two phases: in the first phase, the average annual water transfer capacity is 9.50 billion m^3^, and the water transfer target of the second phase is 13.0 billion m^3^. The first phase of the middle route of the South-to-North Water Diversion Project was completed in December 2014 and began to supply water to the north (Figure 1). After the full operation of the first phase of the middle route of the South-to-North Water Diversion Project, the runoff from the Danjiangkou to Xinglong reaches of the Hanjiang River was reduced by 18.0–25.0%, and the annual average discharge was reduced by about 300 m^3^/s, resulting in the significant reduction in the runoff of the Danjiangkou to Xinglong reaches [24]. Additionally, with the development of cascade power stations in the main stream of the Hanjiang River (including Xinji Dam, Yakou Dam, and Nianpanshan Dam), the flow velocity in the reservoir will decrease after the operation of the reservoir, which will greatly reduce the water environmental capacity of the section from the Danjiangkou to Xinglong reaches of the Hanjiang River [52,53]. Due to the implementation of the Yangtze–Hanjiang Water Diversion Project (Figure 1), the river basins below the Xinglong reservoir area of the Hanjiang River can basically meet the needs of ecological flow [52,53]. Due to the decrease in runoff from the upper reaches and the slowing down of the flow velocity, the water environment in the Danjiangkou to Xinglong reaches of the Hanjiang River is worthy of attention. Meanwhile, the changes of suspended sediment (SS) also need to be explained.

Taking the Huangzhuang Hydrological Station from the Danjiangkou to Xinglong reaches of the Hanjiang River as an example, the runoff and SS concentration decreased from 475 × 10^8^ m^3^ and 1.00 kg/m^3^ in 1950–2010 to 380 × 10^8^ m^3^ and 0.0520 kg/m^3^ in 2018 (Table 2). The annual average grain size of the SS in the middle and lower reaches of the Yangtze River has tended to decrease [24], which may be due to the fact that the fine-grained sediments in the riverbed of the Danjiangkou to Xinglong reaches of the Hanjiang River were washed away. When the riverbed of the Danjiangkou to Xinglong reaches of the Hanjiang River was eroded, the PAEs in the surface sediments of the riverbed would have also been released, which might have led to a gradual decline in the concentration of PAEs in the surface sediments. However, the dynamic characteristics of PAEs in water bodies were not consistent with those of PAEs in sediments. Due to the low runoff, the decrease in SS concentration and thus the decrease in the adsorption capacity of PAEs in sediment, the PAE concentration of the water body in the Danjiangkou to Xinglong reaches of the Hanjiang River may increase in the future.

With the development of cascade hydropower stations in the middle-lower Hanjiang River, it is difficult to reach an equilibrium in a short period of time in the deposition–erosion of the reservoirs, and the coarsening of riverbed sediment caused by dam retention and fine particle scouring will continue to increase in future, especially in the Danjiangkou to Xinglong reaches of the Hanjiang River. Therefore, with the changes of runoff, suspended sediment content and particle size, the effect of the concentration fluctuations of PAEs and other organic toxic pollutants in the middle-lower Hanjiang River will continue to exist and need to be further studied systematically.

## 5. Conclusions

Through field survey, monitoring, and comparisons with historical data and risk assessments, the distribution characteristics and trends of PAEs were studied in surface water and sediment in the middle-lower Hanjiang River, and the following main conclusions were reached.

The patterns of different distributions of PAEs varied substantially along the river without clear trends. PAE monomers were lower than their corresponding water quality threshold values (GB 3838-2002). However, DEHP concentrations in the surface water exceeded the value of the human health AWQC set by the USEPA at some sampling sites, revealing potential pollution risks, as was the case in the Laohekou, Qianjiang, and Hanchuan sections. DBP and DEHP concentrations at some sampling sites in the sediment exceeded their ERLs, which were relatively high in the Qianjiang and Wuhan sections. Compared with other regions at home and abroad, the pollution levels of surface water and sediments in the middle-lower Hanjiang River were relatively low. Higher concentrations and more PAE monomers were detected in the dry season than in the wet season in surface water and sediments. With the reduction in runoff from the upper reaches, the slowing down of the flow velocity, the decrease in SS concentration, and the decrease in PAEs’ adsorption capacity in sediment, the PAE concentration of the water body in the Danjiangkou to Xinglong reaches of the Hanjiang River may increase in the future.

Given that DEHP and DBP are toxic, bioaccumulative, and detected by high frequency and high concentrations, more studies to investigate their levels in organisms and the corresponding toxicity evaluation in biota are also needed. With the increase in microplastics in the environment on the surface, toxic organic pollutants (such as PAEs) in microplastics will also be released into environmental water bodies with changes in environmental conditions. Therefore, the ways in which different environmental factors affect the release of PAEs in microplastics need to be further studied. In addition, long-range atmospheric transport deposition may also be one of the PAE sources in the middle-lower Hanjiang River, which requires further studies to evaluate the impact of this on PAE sources in the Hanjiang River basin.

## Figures and Tables

**Table 1:** AWQC for human health set by the USEPA for PAEs (μg/L).

PAEs	Human Health AQQC for		This Study	
	The Consumption of Water + Organism	The Consumption of Organism Only	(Wet Season) × 10^−3^	(Dry Season) × 10^−3^
DMP	2.00 × 10^3^	2.00 × 10^3^	6.20–361 (mean 45.6)	ND-84.4 (mean 22.7)
DEP	600	600	40.4–422 (mean 130)	128-553 (mean 253)
DBP	20.0	30.0	266–1.21 × 10^3^ (mean 596)	570-1.48 × 10^3^ (mean 1.01 × 10^3^)
BBP	0.100	0.100	ND-17.4 (mean 3.70)	ND-90.6 (mean 25.9)
DEHP	0.320	0.370	85.5–748 (mean 369)	140-861 (mean 427)

**Table 2:** Annual average hydrological parameters at the Huangzhuang hydrological stations in the middle-lower Hanjiang River.

Period	Runoff (10^8^ m^3^)	SS Load (10^8^ t)	SS Concentration (kg/m^3^)	SS Grain Size (μm)
1950–2010	475	0.477	1.00	52.0
2011	513	0.0540	0.104	38.0
2012	433	0.0370	0.0850	44.0
2013	326	0.0150	0.0470	27.0
2014	215	0.00700	0.0340	22.0
2015	364	0.0170	0.0480	51.0
2016	242	0.0130	0.0550	19.0
2017	446	0.0610	0.138	19.0
2018	380	0.0200	0.0520	25.0

## Data Availability

The datasets used and/or analyzed during the current study are available from the corresponding author on reasonable request.

## Abbreviations

PAEs: Phthalic Acid Esters; DMP: dimethyl phthalate; DEP: diethyl phthalate; DBP: di-n-butyl phthalate; BBP: butyl benzyl phthalate; DEHP: di-2-Ethylhexyl phthalate; DNOP: di-n-octyl phthalate; DOC: dissolved organic carbon; TN: total nitrogen; TP: total phosphorus; NH_3_-N: ammonia nitrogen; DO: dissolved oxygen; T: temperature; ORP: oxidation–reduction potential.

## Appendix A

**Table A1:** Geographical information and water quality parameters at the sampling sites from the middle-lower Hanjiang River.

Station	Sampling Sites	Sampling Position	Parameters (Wet Season/Dry Season)			
			pH	DO (mg/L)	T (°C)	ORP (mv)
S1	0.5 km under Danjiangkou Dam	N32°33′24.2”, E111°29′23.9”	8.30/7.80	10.8/10.2	26.2/12.8	71.6/181
S2	Danjiangkou urban section	N32°32′1.77”, E111°30′12.9”	7.60/8.00	9.13/10.5	22.6/9.80	112/197
S3	Hydrological Station of Huangjiagang	N32°30′14.9”, E111°31′2.5”	7.81/7.92	9.32/10.4	23.1/9.41	114/148
S4	Laohekou urban section	N32°21′36.8”, E111°39′46.7”	7.31/7.91	5.02/9.9	27.1/11.3	147/95.1
S5	Qing River	N32°3′51.5”, E112°10′15.9”	6.13/9.60	6.21/10.4	26.4/10.9	122/139
S6	Tangbai River	N32°4′43.3”, E112°12′42.4”	6.61/7.70	7.50/10.6	30.1/8.31	87.6/176
S7	Zhongxiang urban section	N31°11′39.1”, E112°34′27.0”	8.60/7.91	7.92/10.8	26.2/10.3	117/175
S8	Zhupi River	N30°53′26.5”, E112°33′1.29”	8.72/7.54	7.91/9.9	25.5/7.30	124/184
S9	Downstream of Xinglongba gate	N30°36′38.9”, E112°40′47.0”	8.22/8.13	8.62/11.5	25.7/8.72	65.3/155
S10	Qianjiang urban section	N30°30′32.3”, E112°53′9.5”	8.40/7.90	7.52/11.4	24.8/8.61	56.8/183
S11	Xiantao urban section	N30°22′46.5”, E113°27′1.26”	8.21/8.02	7.63/11.7	25.3/8.20	88.6/205
S12	Hanchuan urban section	N30°38′26.8”, E113°50′38.8”	7.74/7.92	5.71/11.2	26.2/9.12	46.2/174
S13	Upstream of Wuhan	N30°35′34.7”, E114°8′51.4”	7.62/7.83	5.11/11.6	27.1/7.70	45.1/198
S14	Midstream of Wuhan	N30°34′13.2”, E114°14′34.5”	7.52/7.84	4.83/11.3	26.7/8.52	63.9/214
S15	Downstream of Wuhan	N30°33′54.1”, E114°17′21.9”	7.61/8.13	4.74/11.1	27.3/8.32	40.8/182

**Table A2:** The concentrations of PAEs in the surface water of the middle-lower Hanjiang River (ng/L).

PAEs	Wet Season				Dry Season			
	Range	Mean ± SD ^a^	Median	DR ^b^ (*n* = 15, %)	Range	Mean ± SD	Median	DR (*n* = 15, %)
DMP	6.20–361	45.6 ± 89.2	15.5	100	ND–84.4	22.7 ± 27.6	9.00	60.0
DEP	40.4–423	130 ± 96.7	103	100	128–553	253 ± 118	196	100
DBP	266–1.21 × 10^3^	596 ± 282	452	100	567–1.48 × 10^3^	1.01 × 10^3^ ± 320.8	1.11 × 10^3^	100
BBP	ND ^c^–17.4	3.70 ± 4.70	2.80	73.3	ND–90.6	25.9 ± 33.0	15.6	80.0
DEHP	85.5–748	369 ± 210	338	100	140–861	427 ± 206	408	100
DNOP	ND–36.0	13.7 ± 10.2	11.3	93.3	ND–162	51.3 ± 44.2	44.9	80.0
∑_6_PAEs	592–2.03 × 10^3^	1.16 × 10^3^ ± 451	1.15 × 10^3^	100	1.31 × 10^3^–2.75 × 10^3^	1.79 × 10^3^ ± 406.1	1.65 × 10^3^	100

^a^ standard deviation. ^b^ detection rate. ^c^ concentration was lower than the LOQ.

**Table A3:** The sampling sites and concentrations of the middle-lower Hanjiang River.

**Wet Season**	**Concentration (ng/L)**						
**Sampling Site**	**DMP**	**DEP**	**DBP**	**BBP**	**DEHP**	**DNOP**	**∑_6_PAEs**
S1	6.20	40.4	266	0.90	266	13.2	592
S2	22.5	104	814	5.60	717	18.6	1.68 × 10^3^
S3	8.40	242	317	0.60	97.1	9.70	675
S4	10.2	171	1.21 × 10^3^	17.4	615	5.80	2.03 × 10^3^
S5	13.9	70.0	407	0.90	152	3.90	648
S6	15.5	147	878	ND	386	9.70	1.43 × 10^3^
S7	11.8	108	628	2.80	245	12.8	1.01 × 10^3^
S8	361	83.6	452	8.10	338	36.0	1.28 × 10^3^
S9	15.4	54.3	824	7.20	748	6.50	1.66 × 10^3^
S10	16.7	94.0	446	ND	265	11.3	832
S11	33.7	103	406	ND	385	34.9	963
S12	27.0	78.3	1.00 × 10^3^	4.20	445	17.8	1.57 × 10^3^
S13	59.6	423	524	4.30	238	16.1	1.26 × 10^3^
S14	12.8	168	411	4.20	548	ND	1.15 × 10^3^
S15	68.6	65.6	362	ND	85.5	10.0	592
**Dry Season**	**Concentration (ng/L)**						
**Sampling Site**	**DMP**	**DEP**	**DBP**	**BBP**	**DEHP**	**DNOP**	**∑_6_PAEs**
S1	51.6	180	1.29 × 10^3^	90.6	516	88.5	2.22 × 10^3^
S2	9.00	196	691	43.2	401	35.9	1.38 × 10^3^
S3	ND	264	913	ND	271	57.2	1.51 × 10^3^
S4	84.4	183	615	ND	409	21.9	1.31 × 10^3^
S5	33.3	372	859	78.2	312	ND	1.65 × 10^3^
S6	ND	438	715	5.90	140	23.0	1.32 × 10^3^
S7	ND	226	1.11 × 10^3^	15.6	228	33.0	1.61 × 10^3^
S8	42.8	128	1.35 × 10^3^	22.7	263	44.9	1.85 × 10^3^
S9	6.70	180	1.38 × 10^3^	87.9	461	85.2	2.20 × 10^3^
S10	13.0	226	1.48 × 10^3^	6.00	861	162	2.75 × 10^3^
S11	ND	180	570	16.1	731	83.9	1.58 × 10^3^
S12	ND	186	579	0.30	722	88.8	1.58 × 10^3^
S13	64.3	171	1.25 × 10^3^	6.10	408	ND	1.90 × 10^3^
S14	35.8	317	1.18 × 10^3^	ND	258	ND	1.79 × 10^3^
S15	ND	553	1.18 × 10^3^	16.0	421	45.0	2.21 × 10^3^

**Table A4:** PAE concentrations in surface water and sediments from various sites in China and abroad.

Phase	Locations	Sampling Time	Sites	Numbers	DBP	DEHP	ΣPAEs	Mean of ΣPAEs	Reference
Water (ng/L)	Humber River, UK	1995–1996	6	— ^d^	—	740–18.0 × 10^3^	—	—	(Long et al., 1998)
	Kaveri River, India	2012	16	6	250	514	313–4.64 × 10^3^	—	(Selvaraj et al., 2015)
	Jiangsu section of the Yangtze River	2004–2005	15	6	105–286	ND–836	178–1.47 × 10^3^	902	(He et al., 2011)
	The mid-lower reaches of the Yellow River	2004	12	5	ND–26.0 × 10^3^	347–31.8 × 10^3^	—	—	(Juan et al., 2006)
	Jiulong River, China	2014	35	6	30.0–1.77 × 10^3^	6.20 × 10^3^–1.24 × 10^3^	3.48 × 10^3^–17.7 × 10^3^	6.12 × 10^3^	(Li et al., 2017)
	Songhua River, China	2011	11	6	1.69 × 10^3^–11.8 × 10^3^	2.26 × 10^3^–11.6 × 10^3^	9.93 × 10^3^–45.6 × 10^3^	19.8 × 10^3^	(Gao et al., 2014)
	Changjiang River Estuary, China	2015	81	16	—	—	180–3.42 × 10^3^	944	(Zhang et al., 2018)
	Three Gorges Reservoir, China	2015	20	6	12.1–724	1.70–394	4.00–1.17 × 10^3^	398	(Lin et al., 2018)
Sediment (ng/g)	Humber River, UK	1995–1996	6	—	—	840–31.0 × 10^3^	—	—	(Long et al., 1998)
	Cochin Estuary, India	2013	15	6	ND–3.65 × 10^3^	6.00–349	44.0–4.01 × 10^3^	2.25 × 10^3^	(Ramzi et al., 2018)
	Changjiang River Estuary, China	2015			—	—	480.0–29.9 × 10^3^	12.9 × 10^3^	(Zhang et al., 2018)
	Songhua River, China	2017	19	6	1.73 × 10^3^–22.5 × 10^3^	4.81 × 10^3^–18.9 × 10^3^	6.83 × 10^3^–36.3 × 10^3^	18.4 × 10^3^	(Wang et al., 2020)
	Three Gorges Reservoir	2015	20	6	82.9–4.05 × 10^3^	10.9–1.11 × 10^3^	177–4.74 × 10^3^	2.23 × 10^3^	(Lin et al., 2018)
	Xijiang River, China	2016	12	6	—	—	21.0–71.0	—	(Tang et al., 2018)

^d^ no data.

**Table A5:** The concentrations of PAEs in sediments of the middle-lower Hanjiang River (ng/g).

PAEs	Wet Season				Dry Season			
	Range	Mean ± SD	Median	DR (*n* = 15, %)	Range	Mean ± SD	Median	DR (*n* = 15, %)
DMP	2.30–22.9	10.8 ± 4.80	11.4	100	11.9–191	49.3 ± 51.9	33.4	100
DEP	34.0–368	132 ± 84.9	107	100	2.00–620	196 ± 1150	183	100
DBP	159–2.12 × 10^3^	921 ± 506	835	100	400–4.33 × 10^3^	2.01 × 10^3^ ± 1.05 × 10^3^	1.87 × 10^3^	100
BBP	ND–21.1	5.40 ± 6.10	2.70	93.3	ND–12.8	5.90 ± 4.30	6.60	80.0
DEHP	580–1.89 × 10^3^	1.11 × 10^3^ ± 400	1.06 × 10^3^	100	341–1.41 × 10^3^	735 ± 359	600	100
DNOP	44.2–417	140 ± 126	81.3	100	ND–232	63.8 ± 72.9	34.8	80.0
∑_6_PAEs	1226.9–3.38 × 10^3^	2.32 × 10^3^ ± 715	2.25 × 10^3^	100	1.12 × 10^3^–6.61 × 10^3^	3.06 × 10^3^ ± 1.46 × 10^3^	3.05 × 10^3^	100

**Table A6:** Correlation coefficients between concentrations of PAEs and water quality indexes (WQI) in water samples of the middle-lower Hanjiang River.

	PAEs	TP	COD_Mn_	Chl-*a*	TN	NH_4_-N	DOC	DBP	DEHP
PAEs	1								
TP	0.003	1							
COD_Mn_	0.010	0.958 **	1						
Chl-*a*	0.127	0.747 **	0.795 **	1					
TN	0.025	0.965 **	0.922 **	0.822 **	1				
NH_4_-N	0.006	0.921 **	0.846 **	0.763 **	0.972 **	1			
DOC	0.814 **	0.214	0.238	0.380	0.253	0.251	1		
DBP	0.906 **	−0.168	−0.120	0.031	−0.156	−0.207	0.737 **	1	
DEHP	0.835 **	−0.174	−0.165	−0.063	−0.131	−0.092	0.647 **	0.667 **	1

** Correlation is significant at the 0.01 level (2-tailed).

**Table A7:** Correlation coefficients between concentrations of PAEs and water quality indexes (WQI) in sediment samples of the middle-lower Hanjiang River.

	PAEs	OM	TN	TP	DBP	DEHP
PAEs	1					
OM	0.700 **	1				
TN	−0.133	0.132	1			
TP	0.234	0.398	0.690 **	1		
DBP	0.768 **	0.370	−0.337	−0.021	1	
DEHP	0.781 **	0.678 **	−0.185	0.159	0.273	1

** Correlation is significant at the 0.01 level (2-tailed).
